# Supplementary material for: DNA methylation in relation to gestational age and brain dysmaturation in preterm infants
Source: Brain Commun. 2022 Mar 8;4(2):fcac056. doi: 10.1093/braincomms/fcac056 (PMC8984700; doi:10.1093/braincomms/fcac056)
Supplement: fcac056_Supplementary_Data [file fcac056_supplementary_data.zip › Supplementary_Material.docx]

Supplementary Material: DNA methylation and brain dysmaturation in preterm infants

Emily N. W. Wheater, Paola Galdi, Daniel L. McCartney, Manuel Blesa^1^, Gemma Sullivan^1^, David Q. Stoye^1^, Gillian Lamb^1^, Sarah Sparrow^1^, Lee Murphy^2^, Nicola Wrobel^2^, Alan J. Quigley^3^, Scott Semple^4,5^, Michael J. Thrippleton^4,6^, Joanna M. Wardlaw^6^, Mark E. Bastin^6^, Riccardo E. Marioni^2^, Simon R. Cox^7^, James P. Boardman^1,6^

**Supplementary Figure 1. Overview of the analysis pipeline.**

*genome-wide significance threshold (Saffari, A. *et al.* *Genet. Epidemiol.* 2018).

**Differentially methylated region is a region containing two or above sites separated by ≤500 bp with EWAS analysis p≤0.05 and methylation changes in a consistent direction


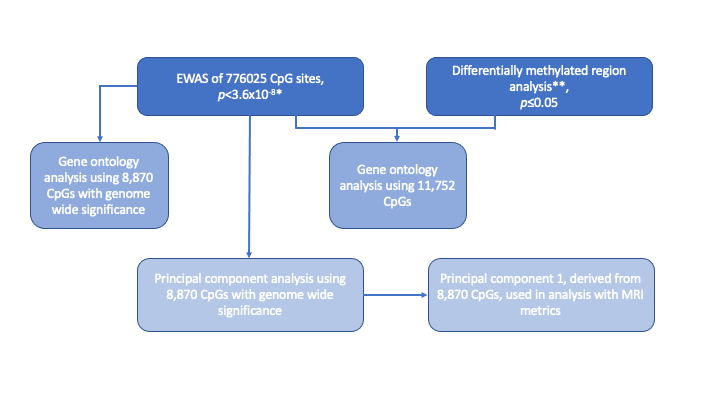


**Supplementary Figure 2. Participant and data flow. dMRI, diffusion magnetic resonance imaging; FA, fractional anisotropy; MD, mean diffusivity; NODDI, neurite orientation dispersion and density imaging; NDI, neurite density index; ODI, orientation dispersion index.**

**
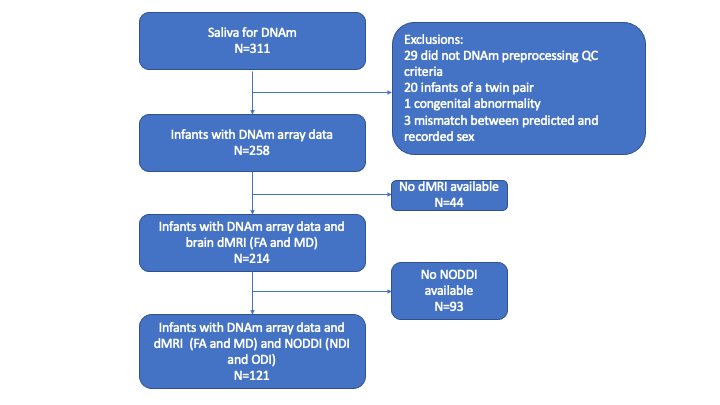
**

**Supplementary Figure 3. Quantile-Quantile (QQ) plot for EWAS results. Genomic inflation factor: 1.72.**

**
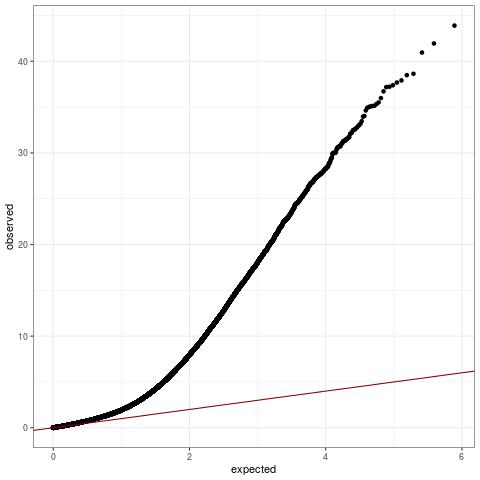
**

**Supplementary Figure 4**. Scatter plots showing the relationship between the beta values of top ten most significant CpG probes and gestational age at birth in weeks, with 95% confidence intervals.


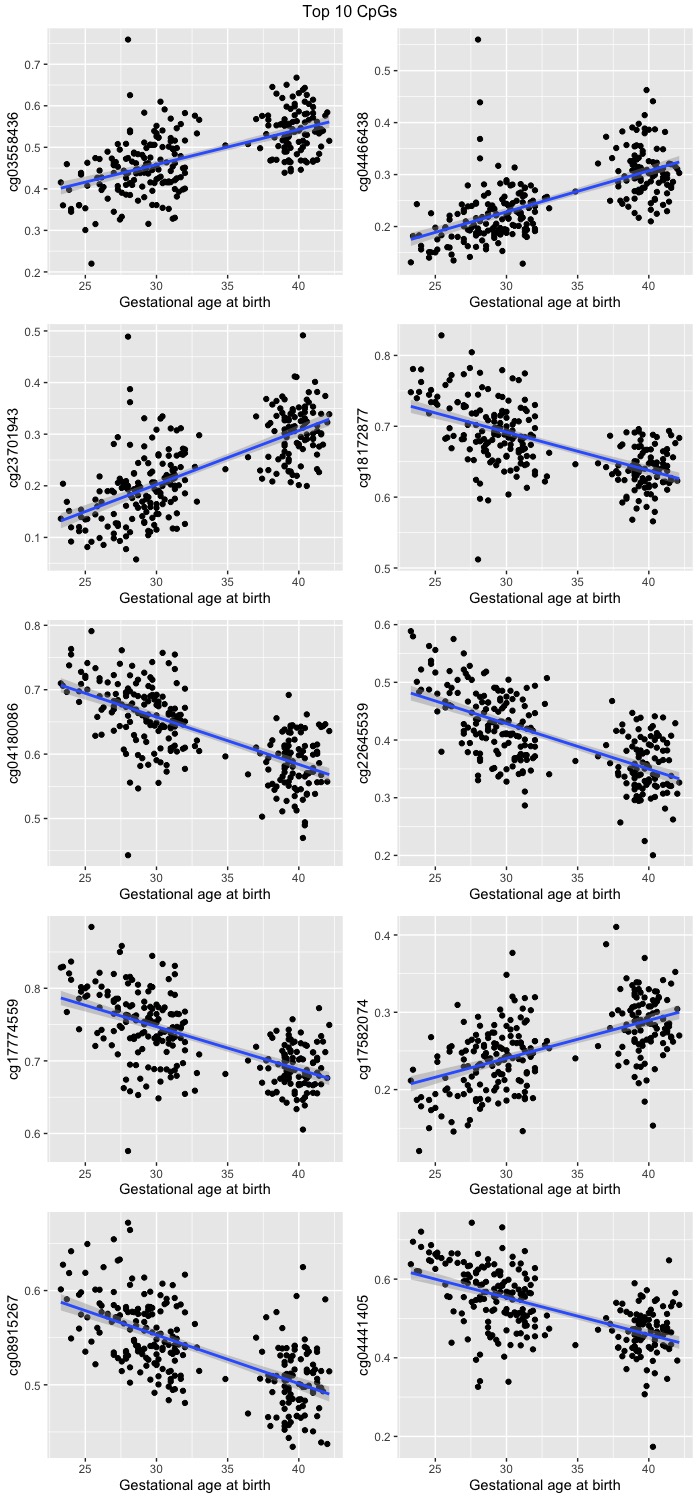


**Supplementary Figure 5.** Batch effects have been successfully removed from the residualised DNAm data based on the top 2 PCs derived from 8,870 CpG probes that reached genome-wide significance.


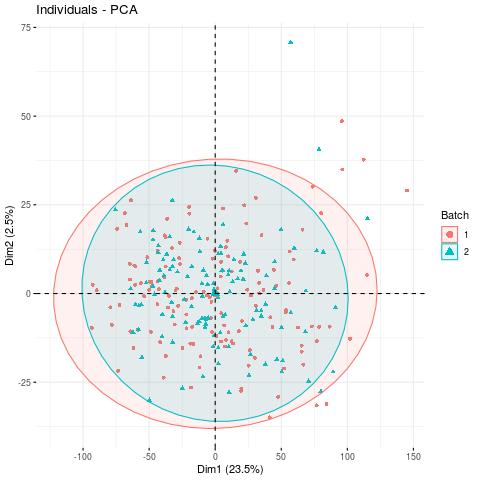


**Supplementary Table 1.** Demographics of participants with both PS (FA and MD) metrics and DNAm data

|  | **Preterm Infants**  (n=127) | **Term Infants**  (n=87) |
| --- | --- | --- |
| Gestational age at birth/weeks (range) | 29.14  (23.28 – 34.86) | 39.74  (36.43 – 42.14) |
| Gestational age at scan/weeks (range) | 40.44  (37.70-44.29) | 42.25  (39.84- 47.14) |
| Birth weight/g  (range) | 1184  (500 – 2100) | 3489  (2410 -4670) |
| Birth weight z-score  (range) | -0.2476  (-3.1324 – 1.5809) | 0.4521  (-2.2952 – 2.9620) |
| Maternal folate supplementation in pregnancy (%) | 110  (87) | 76  (87) |
| Sex: Female (%) | 63 (50) | 39 (45) |
| Maternal age (years) | 31.0  (17-44) | 33.9  (23 – 45) |
| Maternal tobacco smoker in pregnancy (%) | 24 (19) | 0 (0) |

**Supplementary Table 2.** Demographics of participants with both PSNDI metric and DNAm data

|  | **Preterm Infants**  (n=64) | **Term Infants**  (n=57) |
| --- | --- | --- |
| Gestational age at birth/weeks (range) | 29.28  (23.43 – 32.00) | 39.71  (36.43 – 42.14) |
| Gestational age at scan/weeks (range) | 40.44  (38.29-44.29) | 42.25  (40.00- 47.14) |
| Birth weight/g  (range) | 1266  (500 – 2100) | 3502  (2410 -4560) |
| Birth weight z-score  (range) | -0.1083  (-3.1324 – 1.5809) | 0.4996  (-2.2952 – 2.5703) |
| Maternal folate supplementation in pregnancy (%) | 50  (78) | 47  (82) |
| Sex: Female (%) | 25 (39) | 25 (44) |
| Maternal age (years) | 31.2  (21-44) | 34.2  (23-44) |
| Maternal tobacco smoker in pregnancy (%) | 11 (17) | 0 (0) |
