# Supplementary material for: DNA methylation in relation to gestational age and brain dysmaturation in preterm infants
Source: Brain Commun. 2022 Mar 8;4(2):fcac056. doi: 10.1093/braincomms/fcac056 (PMC8984700; doi:10.1093/braincomms/fcac056)
Supplement: fcac056_Supplementary_Data [file Original Submission – Decision Letter.pdf › Original Submission – Decision Letter.pdf]

Original Submission – Decision Letter

18-Aug-2021

Dear Dr. Boardman,

Thank you for your recent submission BRAINCOM-2021-126 entitled "DNA methylation and brain dysmaturation in preterm infants" to Brain Communications.

Your manuscript has been reviewed, and while the reviewers and editorial team appreciate many aspects of the paper, they raise some concerns that you will need to address in order for the paper to be considered for publication.

If you can address these concerns, I invite you to submit a revised manuscript and a response to each of the reviewers' comments. The reviewer(s)' comments are included at the bottom of this message.

In addition to the reviewers' comments, please note our editorial comments added below to comply with our requirements for presentation of scientific data at Brain Communications.

Please format your revised manuscript according to our Instructions to Authors https://academic.oup.com/braincomms/pages/General_Instructions – a summary of key points is attached to help with this.

To revise your manuscript, click the link below or log into https://mc.manuscriptcentral.com/braincom and enter your Author Centre, where you will find your manuscript title listed under "Manuscripts with Decisions." Under "Actions," click on "Create a Revision." Your manuscript number will be appended to denote a revision.

*** PLEASE NOTE: This is a two-step process. After clicking on the link, you will be directed to a webpage to confirm. ***

https://mc.manuscriptcentral.com/braincom?URL_MASK=8dfeaf14706a46fc8f5738773705c67a

Please upload a revised manuscript with changes tracked with track changes or in coloured text.

When submitting your revised manuscript, you will be asked to respond to the reviewer(s) comments. Please be as specific as possible in your response to all of the comments.

The editorial team at Brain Communications aims to increase transparency in scientific publishing and to demonstrate the value that peer review brings to the scientific process. Please therefore consider whether you are willing to have reviewer(s)' comments on your paper and your response published as supplementary data with the paper if it is accepted. This is entirely optional and will only be done if both the authors and reviewers agree. While submitting your revision there will be an opportunity to review or update the answer you gave previously, if necessary.

IMPORTANT: Your original files are available to you when you upload your revised manuscript. Please delete any redundant files before completing the submission.

Because we are trying to facilitate timely publication of manuscripts submitted to Brain Communications, we would appreciate your revised manuscript as soon as possible. If it is not possible for you to resubmit within 3 months, the option to submit a revision will expire, and we would then consider your revised paper as a new submission. However, if you need a little more time, please get in touch and we can extend this deadline for you.

Once again, thank you for submitting your manuscript to Brain Communications and I look forward to receiving your revision.

Best wishes

Dr. Alberto Lleo

Associate Editor, Brain Communications

Reviewer(s)' Comments to Author:

Reviewer: 1

Comments to the Author
Wheater et al. investigated the link between preterm birth, DNA methylation and brain dysmaturation in a sample of up to 258 infants. The authors report a link between preterm birth and DNAm; preterm birth and some (but not all) MRI measures; and DNAm and some (not all) MRI measures.

The manuscript is well written and insightful.

I have the following comments:
1) How to their EWAS results compare to gestational age EWAS results, based on cord blood, in terms of direction of effect and proportion of associated CpG sites (vs all tested CpGs)? Did previous studies also report 50/50 in-/decreases in DNAm and a signal in about 1% of all sites?
2) The study design is a little complex. For example, it is not very clear when exactly DNAm and MRI measures were obtained (at birth, shortly after; what is phase 1 and phase 2?). Also, how many participants were lost of which stage and how large exactly is the overlap between MRI and DNAm? I suggest a study design flowchart to make this clear.
3) How many batches were there and how many scanner (was it a different scanner for phase 1 and 2 and if so, why)?
4) How many SVs were included in the final analyses? Why did the authors correct for birth weight, which is highly collinear with / part of the preterm birth phenotype? Is it meaningful to look at preterm birth, while removing any effect linked to birthweight given that these two traits probably correlated quite strongly with each other (in a meaningful way, not in a confounding way)? What are the strengths and limitations of doing this; and how does it compare to previous studies on gestational age and DNAm?
5) As with the study design, the statistical analysis flow and results could be clearer. It seems to follow something like
a. EWAS of preterm birth (once at $p<3.6x10-8$ and once Bonferroni-corrected, which corresponds to which threshold exactly?);
b. DMR on nominal hits (not EWAS hits from the step before);
c. gene-set based on EWAS hits and then again on EWAS+DMR results;
d. PCA of EWAS hits (based on $p<3.8x10-8$?), but excluding DMRs;
e. PC1 on gestational age (but not preterm vs control, which was used before?)…
There seems to be some inconsistency in what exactly got followed forward at which step and based on which threshold. Again, I think a simple figure would quite nicely clarify this.
6) Could the authors comment on how heavily influenced the assessed MRI measures are by intracranial volume? Is this something that needs to be controlled for?
7) Table 5: I don't think 'gestational age at scan' makes sense. Just 'age at scan'? Also, is this done in the whole sample or per group? And is 'gestational age' a proxy for preterm-vs-control? Why not use this group variables rather than a continuous measure?
8) I feel like most figures should show regression lines per group, not overall. There are clearly two very different groups shown and fitting a line overall, across both groups does not make sense to me. Please explain or change.

Minor comments:
1) Minor edit: please add 'were' to Page 6, line 28 ('watermelon and minfi >were<…'); and change 'p value' to 'p-value'.
2) 'imbalance of birthweight or sex' – please clarify with respect to which other variable or group.
3) Minor edit: '…CpG sites were significant >at< $p<0.05$' -> please add the 'at'.
4) Is it fair to refer to controls as 'healthy'? Is being preterm by definition an illness or were all pre-term infant ill?
5) Can the authors add to table 1 whether or not groups differed in any of the listed variables?
6) Table 2-3 need a measure of SE or 95% CI.
7) Table 4 needs some information on number of included CpGs/genes contributing to these pathways
8) I really appreciated the discussion of a potential mediation analysis in the limitation section, which I felt was needed.

Reviewer: 2

Comments to the Author

Wheater et al conducted a study among 258 neonates, embedded within the Theirworld Edinburgh Birth Cohort, a longitudinal study. The first aim was to determine whether the combined exposures of preterm birth followed by postnatal exposures during neonatal intensive care are associated with neonatal saliva DNA methylome at term equivalent age. The second aim was to explore whether saliva-specific DNA methylation signatures of preterm birth explains variance in measures of white matter microstructure assessed with MRI. Gestational age at birth analyzed as a continuous variable was associated with widespread differential methylation at 8,870 CpG probes and 1,767 DMRs. Associations between DNA methylation and specific MRI measurements suggest that variations in DNA methylation could contribute to white matter dysconnectivity commonly seen in preterm infants.

This manuscript is well-written and easy to follow. It is of particular interest as the analyses are done in infant saliva samples. Such samples are relatively easy to collect, which makes the work relevant for future studies to detect and/or monitor DNA methylation changes over time. Next to this, MRI acquisition in infants is a robust image phenotype.

I have a number of questions and suggestions to the authors.

1. On a first read, the manuscript seems to describe associations of gestational age with DNA methylation. However, what is in fact assessed is "DNA methylation status at term equivalent age". As the authors mention in the conclusion section, in preterm born neonates this would comprise more than gestational age at birth, since it also takes into account the postnatal exposures during neonatal intensive care. I suggest to make this clearer throughout the manuscript and to be clear on which exposure is being tested, namely being born preterm plus the following treatment.
2. Title: the title mainly reflects the second aim of the paper. Could the authors rephrase the title to better reflect the full content?
3. Methods: Please add how gestational age was defined/assessed.
4. Methods: Could the authors add information on the causes of prematurity in their population.
5. Methods: The paper would benefit from a flowchart of the study population.
6. Methods: The authors describe 2 phases of MRI acquisition. Please explain the reason for the second phase (new hospital procedures, based on indication, …) and also show the distribution of the neonates in both phases regarding gestational age at birth.
7. Methods, paragraph Gene set testing: It is not clear from the description that the gene set enrichment analysis was first done on the hits from the EWAS and then on those from the EWAS plus those from the DMR analysis. Please clarify.
8. Results, first paragraph: Please describe in the text the gestational age range for both preterm and a term neonates.
9. Results, first paragraph: The high smoking prevalence among preterm neonates is remarkable. Could the authors comment on this?
10. Discussion: One of the manuscript's main limitations is that there is no attempt at replication. Did the authors search for a study with these data that might be able to replicate the findings? The manuscript would be strengthened by adding replication, if possible. If not available, please comment on this in the discussion section.
11. Discussion: I agree that the most likely direction of effect is in line with the hypothesis of the authors. However, did the authors consider the direction of effects could be turned around? In others words, fetal differential DNA methylation induces prematurity? Then, DNA methylation would not mediate the assessed associations with brain development.
12. Discussion: "the most significant DMR…decreases throughout development." In this line 2 genes are mentioned, but only 1 is further discussed. Please include the other gene as well for completeness.
13. Discussion, page 13 line 19, paragraph concerning "probes that demonstrated the largest magnitude of effect". The authors describe their findings in terms of hypermethylated and hypomethylated genes. However, it is (very likely) not a matter of a complete gene being hyper- or hypomethylated, but rather probes in a certain part of these genes (e.g. promoter, gene body) showing differential methylation in relation to gestational age and thus potentially having differential effects on gene expression. Please describe more specifically.
14. Discussion, page 14 line 46: The authors describe that correcting for the lambda may be overly conservative. Although this is indeed mentioned in the reference cited (Van Iterson et al), this reference also argues in favor of correcting for the inflation using a novel method proposed in the paper. Did the authors consider using this method for correction of inflation and if not, why not?

Editorial Office comments:

- for all the plots, please, report in the corresponding figure legend the test used and relevant test statistic values alongside p values (for example F or t values for ANOVAs and t-tests).

-Please supply a graphical abstract. A graphical abstract is a visual representation of the central finding or methodology of the paper. It will be displayed in the table of contents. Authors should strive to make the graphical abstract informative, interesting, visually appealing, and straightforward (no caption needed, no abbreviations except gene names). Please upload as a separate high resolution image file (tiff preferred). The technical requirements for the graphical abstracts are:

--size of the submitted image: 1200 pixels square at 300dpi

--font of the text: Arial 12-16 point (smaller fonts will not be legible online)
