# Supplementary material for: DNA methylation in relation to gestational age and brain dysmaturation in preterm infants
Source: Brain Commun. 2022 Mar 8;4(2):fcac056. doi: 10.1093/braincomms/fcac056 (PMC8984700; doi:10.1093/braincomms/fcac056)
Supplement: fcac056_Supplementary_Data [file Original Submission-Authors Response.pdf › Original Submission-Authors Response.pdf]

We thank the reviewers for their comments. We have responded point by point below. Changes to the manuscript are highlighted in yellow.

Reviewer: 1

Comments to the Author
Wheater et al. investigated the link between preterm birth, DNA methylation and brain dysmaturation in a sample of up to 258 infants. The authors report a link between preterm birth and DNAm; preterm birth and some (but not all) MRI measures; and DNAm and some (not all) MRI measures.

The manuscript is well written and insightful.

Response 1. Thank you for commenting on new insights reported in this manuscript.

I have the following comments:
1) How to their EWAS results compare to gestational age EWAS results, based on cord blood, in terms of direction of effect and proportion of associated CpG sites (vs all tested CpGs)? Did previous studies also report 50/50 in-/decreases in DNAm and a signal in about 1% of all sites?

Response 2. The results are comparable with gestational age (GA) EWAS results from umbilical cord blood. In meta-analysis Merid *et al* reported 8899 CpGs enriched out of ~450,000 (2%), and CpGs associated with GA had both negative (60%) and positive (40%) directions of effect (Genome Med 2020). In the study by Bohlin et al, which investigated the predictive value of cord blood DNAm for GA, 7.19% of 399,364 autosomal probes assessed differed: 16,190 (56.4%) were hypo- methylated and 12,528 (43.6%) were hyper-methylated (Genome Biol 2016). Another relevant study that is closely related to the point raised by the reviewer is by Spiers et al, which reported both hypo- (56.4%) and hyper-methylation (43.6%) in fetal brain tissue (Genome Res 2015).

We have added the following contextual information to the introduction (paragraph 3):

"A meta-analysis investigating DNAm from umbilical cord blood identified widespread differential methylation associated with GA at birth (across the range 27-42 weeks) affecting 2% of sites and involving both hypo- and hyper-methylation, as measured on the Illumina 450k array.[34]"

We have discussed similarities to these studies in paragraph 2 of the discussion (233 probes associated with GA in our study were also differentially methylated in the cord blood data reported by Merid et al). We have also suggested possible explanations for differences across studies, which are expected because of methodological (tissue types, arrays) and clinical (population characteristics, timing of sampling) heterogeneity.

2) The study design is a little complex. For example, it is not very clear when exactly DNAm and MRI measures were obtained (at birth, shortly after; what is phase 1 and phase 2?). Also, how many participants were lost of which stage and how large exactly is the overlap between MRI and DNAm? I suggest a study design flowchart to make this clear.

Response 3. We have made the following clarifications in methods:

"Saliva obtained at term equivalent age, on the same day as MRI acquisition, was collected in Oragene OG-575 Assisted Collection kits…" This information was in the MRI sub-section of methods but we agree flow is improved by describing it in the sub-section 'DNA extraction and methylation measurement.'

Phase 1 and phase 2 describe the scanner used. This nomenclature does not add value over and above the scanner descriptions so we have removed it. Please also see response 4 and 24.

We have added a chart of participant flow to supplementary material (Supplementary Fig 2). Thank you for this suggestion.

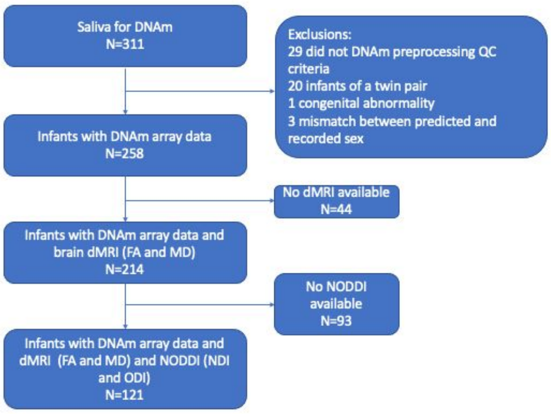

Supplementary Figure 2. Participant and data flow. dMRI, diffusion magnetic resonance imaging; FA, fractional anisotropy; MD, mean diffusivity; NODDI, neurite orientation dispersion and density imaging; NDI, neurite density index; ODI, orientation dispersion index.

3) How many batches were there and how many scanner (was it a different scanner for phase 1 and 2 and if so, why)?

Response 4. DNAm was processed in two batches. Batch was included in the surrogate variable analysis as a potential confounder (see 'Statistical analysis' section of methods) and we found no evidence for a batch effect influencing the association between differential DNAm and white matter microstructure (see supplementary Fig. 5).
Two MRI systems were used: a MAGNETOM Verio 3T for 93 participants and a MAGNETOM Prisma 3T for 121 infants. This is because of a change in scanner facility part way through data collection, which took 4 years due to the rare nature of the study population. Because two scanners were used, we chose a method to characterise white matter microstructure that is robust for multi-site acquisitions (Baykara et al Ann Neurol 2016, PMID27518166), and we included MRI scanner as a covariate in models investigating associations between DNAm and PSFA/PSMD (see methods sub-section 'Linear regression between DNAm and PS metrics').

4) How many SVs were included in the final analyses? Why did the authors correct for birth weight, which is highly collinear with / part of the preterm birth phenotype? Is it meaningful to look at preterm birth, while removing any effect linked to birthweight given that these two traits probably correlated quite strongly with each other (in a meaningful way, not in a confounding way)? What are the strengths and limitations of doing this; and how does it compare to previous studies on gestational age and DNAm?

Response 5. 17 SVs were included in the analysis. This information is included in the 'Statistical analysis' section of methods. We corrected for birth weight z-score, not birth weight itself. Birth-weight z score is normalised for gestational age, and as such is used to define fetal growth restriction which is an independent risk factor for atypical brain development irrespective of GA at birth.

5) As with the study design, the statistical analysis flow and results could be clearer. It seems to follow something like
a. EWAS of preterm birth (once at p<3.6x10-8 and once Bonferroni-corrected, which corresponds to which threshold exactly?);
b. DMR on nominal hits (not EWAS hits from the step before);
c. gene-set based on EWAS hits and then again on EWAS+DMR results;
d. PCA of EWAS hits (based on p<3.8x10-8?), but excluding DMRs;
e. PC1 on gestational age (but not preterm vs control, which was used before?)…
There seems to be some inconsistency in what exactly got followed forward at which step and based on which threshold. Again, I think a simple figure would quite nicely clarify this.

Response 6. The overall a-e sequence that the reviewer lists is correct, although we agree with them that clarity about the analysis pipeline and thresholding could be improved. Specifically, (a) a genome-wide significance threshold of 3.6×10^{-8} was used (Saffari et al Genet Epidemiol 2018), not Bonferroni; (b) DMR was performed on all CpGs; (c) correct; (d) correct; (e) correct. Please be aware that gestational age rather than a binary variable of preterm/term has been used throughout the manuscript. We have edited the manuscript to make these points clearer and have added the following figure to supplementary material:

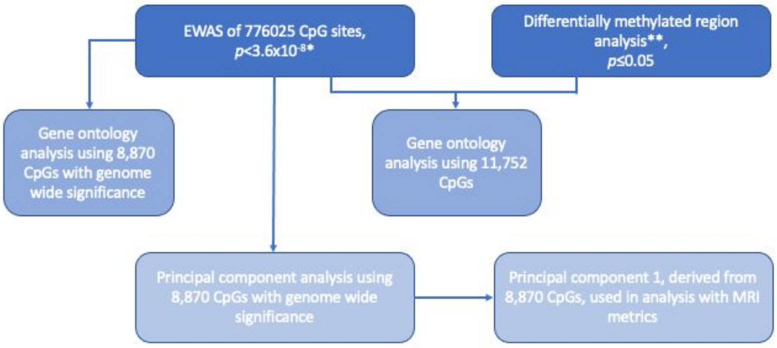

Supplementary Fig 1. Overview of the analysis pipeline.
***genome-wide significance threshold (Saffari, A. *et al. Genet. Epidemiol.* 2018)**
****Differentially methylated region is a region containing two or above sites separated by ≤500 bp with EWAS analysis p ≤ 0.05 and methylation changes in a consistent direction**

We have clarified as follows in the 'Gene set testing' sub-section Methods:
"We performed an analysis that included those sites that reached genome-wide significance in EWAS, and a second analysis that also incorporated those that contributed to differentially methylated regions."

6) Could the authors comment on how heavily influenced the assessed MRI measures are by intracranial volume? Is this something that needs to be controlled for?

Response 7. We did not adjust for brain volume or intracranial volume for two reasons: first, neonatal WM microstructural parameters are not known to be influenced by brain volume; second, we and others have previously shown that total brain volume at term equivalent age is an unreliable marker of prematurity (Boardman et al Ann Neurol 2007). We agree with the reviewer that consideration of adjustment of total brain/ICV may be appropriate in study designs that include regional volumetric comparisons but that was not the case in this study.

7) Table 5: I don't think 'gestational age at scan' makes sense. Just 'age at scan'? Also, is this done in the whole sample or per group? And is 'gestational age' a proxy for preterm-vs-control? Why not use this group variables rather than a continuous measure?

Response 8. We have edited Table 5, as requested. The term 'GA at scan' was used previously because there are different systems for describing postnatal age (corrected, chronological); however, for the purpose of this table, we agree that 'age at scan' suffices and is perhaps less confusing to a general readership.
The analysis was carried out in the whole group. Gestational age is not a proxy for preterm-vs-control; preterm and term birth are both defined by the length of gestation. We selected gestational age at birth as the independent variable due to the dose-dependent relationship between neuroanatomical variation and neurodevelopmental impairment with gestational age, and because previous work (e.g. Merid et al. Genome Med 2020, Spiers et al. Genome Res 2015 and Bohlin et al. Genome Biol 2016) indicate that DNAm is dynamic across the whole gestational age range. Also, using a continuous measure has more power as information is not lost by dichotomising.

8) I feel like most figures should show regression lines per group, not overall. There are clearly two very different groups shown and fitting a line overall, across both groups does not make sense to me. Please explain or change.

Response 9. Please see response 8. We plotted overall regression lines because this reflects the analyses whereby gestational age and not preterm/control group assignment was the independent variable.

Minor comments:
1) Minor edit: please add 'were' to Page 6, line 28 ('watermelon and minfi >were<…'); and change 'p value' to 'p-value'.

Response 10. Done.

2) 'imbalance of birthweight or sex' – please clarify with respect to which other variable or group.

Response 11. This refers to the characteristics of the other twin of the pair, i.e. the random removal of a twin pair did not inadvertently remove the female twin or a lower birth weight twin. We have clarified this in paragraph 1 of the results as follows: "After random removal of one twin from each set, there was no evidence of imbalance for birthweight between the twins that were removed and the twins that remained in the sample (*t*=-0.157, *p*=0.88), or for sex (Chi=0.417, *p*=0.52)."

3) Minor edit: '…CpG sites were significant >at< p<0.05' -> please add the 'at'.

Response 12. Done

4) Is it fair to refer to controls as 'healthy'? Is being preterm by definition an illness or were all pre-term infant ill?

Response 13. We have removed the reference to healthy and used 'term' instead.

5) Can the authors add to table 1 whether or not groups differed in any of the listed variables?

Response 14. We have inserted a column showing *p*-values for analyses of group difference (*t*-test for continuous normally distributed variables and Chi square for proportions).

6) Table 2-3 need a measure of SE or 95% CI.

Response 15. We have added a column for SE to Tables 2 and 3.

7) Table 4 needs some information on number of included CpGs/genes contributing to these pathways

Response 16. We have inserted a column into Table 4 that shows the number of probes that were associated with GA, compared with all probes in the ontology.

8) I really appreciated the discussion of a potential mediation analysis in the limitation section, which I felt was needed.

Response 17. Thank you.

Reviewer: 2

Comments to the Author
Wheater et al conducted a study among 258 neonates, embedded within the Theirworld Edinburgh Birth Cohort, a longitudinal study. The first aim was to determine whether the combined exposures of preterm birth followed by postnatal exposures during neonatal intensive care are associated with neonatal saliva DNA methylome at term equivalent age. The second aim was to explore whether saliva-specific DNA methylation signatures of preterm birth explains variance in measures of white matter microstructure assessed with MRI. Gestational age at birth analyzed as a continuous variable was associated with widespread differential methylation at 8,870 CpG probes and 1,767 DMRs. Associations between DNA methylation and specific MRI measurements suggest that variations in DNA methylation could contribute to white matter dysconnectivity commonly seen in preterm infants.
This manuscript is well-written and easy to follow. It is of particular interest as the analyses are done in infant saliva samples. Such samples are relatively easy to collect, which makes the work relevant for future studies to detect and/or monitor DNA methylation changes over time. Next to this, MRI acquisition in infants is a robust image phenotype.

Response 18. Thanks for noting the interest of the data, robustness of the MRI phenoptype, and for comments on manuscript structure.

I have a number of questions and suggestions to the authors.
1. On a first read, the manuscript seems to describe associations of gestational age with DNA methylation. However, what is in fact assessed is "DNA methylation status at term equivalent age". As the authors mention in the conclusion section, in preterm born neonates this would comprise more than gestational age at birth, since it also takes into account the postnatal exposures during neonatal intensive care. I suggest to make this clearer throughout the manuscript and to be clear on which exposure is being tested, namely being born preterm plus the following treatment.

Response 19. As noted by the reviewer, we have made the point that sampling was at term equivalent age, so incorporates the allostatic load of preterm birth *and* NICU care in the methods and discussion. We have clarified this point further at suitable points in the abstract, introduction (statement of hypotheses) and results.

2. Title: the title mainly reflects the second aim of the paper. Could the authors rephrase the title to better reflect the full content?

Response 20. We have tried to reflect the first aim (to determine whether preterm is associated with variation in the methylome) more obvious by amending the title to: "Differential DNA methylation and brain dysmaturation in preterm infants." This change remains within the 100 character limit required by the journal. We would be happy to consider an alternative if the reviewer/editor thinks it necessary.

3. Methods: Please add how gestational age was defined/assessed.

Response 21. We have added "…based on first trimester ultrasound…" to the participants sub-section of methods.

4. Methods: Could the authors add information on the causes of prematurity in their population.

Response 22. The cause of preterm birth is often unknown and is usually multi-factorial (Goldenberg RL, Lancet 2008). Therefore, it is not possible to state the causes of preterm birth with certainty in unselected cohort studies. We opted to report maternal characteristics that have been associated with differential DNAm in offspring.

5. Methods: The paper would benefit from a flowchart of the study population.

Response 23. Done – see response 3.

6. Methods: The authors describe 2 phases of MRI acquisition. Please explain the reason for the second phase (new hospital procedures, based on indication, …) and also show the distribution of the neonates in both phases regarding gestational age at birth.

Response 24. Please see response 4 to reviewer 1 which describes change in access to MRI facility during the study. The range of GA at birth was similar for infants scanned on each MRI scanner and unlikely to be clinically significant: for the Verio, the mean GA (range) of infants was 32.28 weeks (23.28-41.86); for the Prisma it was 34.0 weeks (23.43-42.14).

7. Methods, paragraph Gene set testing: It is not clear from the description that the gene set enrichment analysis was first done on the hits from the EWAS and then on those from the EWAS plus those from the DMR analysis. Please clarify.

Response 25. We have now clarified this. Please see Response 6 and new supplementary Figure 1.

8. Results, first paragraph: Please describe in the text the gestational age range for both preterm and a term neonates.

Response 26. Done.

9. Results, first paragraph: The high smoking prevalence among preterm neonates is remarkable. Could the authors comment on this?

Response 27. It is estimated that around 10% of British women are smokers at the time of delivery (https://www.nuffieldtrust.org.uk/resource/smoking-in-pregnancy), and in Scotland the proportion of women reporting smoking at the time of booking their pregnancy is estimated to be 14.6% (https://www.scotpho.org.uk/behaviour/tobacco-use/data/maternal-smoking/). In our study population, 19% of the preterm mothers reported smoking at booking, versus 2% of mothers of term infants (Table 1). We expected the prevalence of smoking to be higher among mothers of preterm infants because smoking during pregnancy is a cause of adverse pregnancy outcomes including preterm birth, and smoking is also linked to deprivation. For this reason, we included smoking as a covariate in the analysis - see statistical methods sub-section 'Epigenome-wide association analyses') and results.

10. Discussion: One of the manuscript's main limitations is that there is no attempt at replication. Did the authors search for a study with these data that might be able to replicate the findings? The manuscript would be strengthened by adding replication, if possible. If not available, please comment on this in the discussion section.

Response 28. We are not aware of available data from other cohorts that would enable replication, although replication could be possible in future using data being collected currently (personal correspondence). We have discussed this important issue in the Discussion:

"…This could be addressed by out-of-sample validation, which will require other neonatal cohorts with both saliva methylome and dMRI data. Cohorts, with such data, that recruit neonates from across the gestational age continuum would also provide valuable replication of analyses described here."

11. Discussion: I agree that the most likely direction of effect is in line with the hypothesis of the authors. However, did the authors consider the direction of effects could be turned around? In others words, fetal differential DNA methylation induces prematurity? Then, DNA methylation would not mediate the assessed associations with brain development.

Response 29. We agree, and have added the following to the Discussion: "However, some of the DNAm signature may be related to causes of prematurity that operate in fetal life, such as infection or pre-eclampsia. We cannot rule out that such changes are involved in the aetiology of preterm birth, which might preclude them from mediating specific associations with brain development."

12. Discussion: "the most significant DMR…decreases throughout development." In this line 2 genes are mentioned, but only 1 is further discussed. Please include the other gene as well for completeness.

Response 30. Thank you, we have added information about *BLCAP* to the paragraph as follows:

"The most significant DMR mapped to a site encoding two genes: *NNAT* and *BLCAP. NNAT* encodes the neural fate initiator neuronatin, the expression of which decreases throughout development [65]; there was a positive association with increasing gestational age at birth. Hypomethylation of *NNAT* is associated with a corresponding increase in expression of neuronatin.[66] *BLCAP* encodes the bladder cancer associated protein. This is a tumour suppressor that induces apoptosis, with high expression in brain and b lymphocytes. The candidacy of this locus, encoding *NNAT* and *BLCAP,* as a region of interest whose expression may be modified by perinatal exposures is supported by previous EWAS of gestational age, maternal body mass index, maternal smoking, and schizophrenia.[22,29,62,67–70]. In addition, *BLCAP* was found to have reduced methylation in placental samples from women who had pre-eclampsia. [71]

13. Discussion, page 13 line 19, paragraph concerning "probes that demonstrated the largest magnitude of effect". The authors describe their findings in terms of hypermethylated and hypomethylated genes. However, it is (very likely) not a matter of a complete gene being hyper- or hypomethylated, but rather probes in a certain part of these genes (e.g. promoter, gene body) showing differential methylation in relation to gestational age and thus potentially having differential effects on gene expression. Please describe more specifically.

Response 31. We agree this is an important clarification; we have specified effects are seen at probes within genes, rather than complete genes.

14. Discussion, page 14 line 46: The authors describe that correcting for the lambda may be overly conservative. Although this is indeed mentioned in the reference cited (Van Iterson et al), this reference also argues in favor of correcting for the inflation using a novel method proposed in the paper. Did the authors consider using this method for correction of inflation and if not, why not?

Response 32. Van Iterson et al suggest a correction for inflation that works by adjusting the p-values. We have instead used a stringent p-value threshold for genome wide significance (as suggested by Saffari et al) as the cut-off for EWAS, and reported the inflation for interest. Of note, the inflation that we observed in EWAS analysis was similar to that observed for some of the contributing studies to the meta-analysis of gestational age reported by Merid et al.

Editorial Office comments:

- for all the plots, please, report in the corresponding figure legend the test used and relevant test statistic values alongside p values (for example F or t values for ANOVAs and t-tests).

Response 33. We have now done this.

-Please supply a graphical abstract. A graphical abstract is a visual representation of the central finding or methodology of the paper. It will be displayed in the table of contents. Authors should strive to make the graphical abstract informative, interesting, visually appealing, and straightforward (no caption needed, no abbreviations except gene names). Please upload as a separate high resolution image file (tiff preferred). The technical requirements for the graphical abstracts are:
--size of the submitted image: 1200 pixels square at 300dpi
--font of the text: Arial 12-16 point (smaller fonts will not be legible online)

Response 34. We have uploaded a graphical abstract.
