# Supplementary material for: DNA methylation in relation to gestational age and brain dysmaturation in preterm infants
Source: Brain Commun. 2022 Mar 8;4(2):fcac056. doi: 10.1093/braincomms/fcac056 (PMC8984700; doi:10.1093/braincomms/fcac056)
Supplement: fcac056_Supplementary_Data [file Revision 1 – Decision Letter.pdf › Revision 1 – Decision Letter.pdf]

Revision 1 – Decision Letter

09-Nov-2021

Dear Dr. Boardman,

Thank you for your recent submission BRAINCOM-2021-126.R1
entitled "Differential DNA methylation and brain dysmaturation in preterm infants" to Brain Communications.

Your manuscript has been reviewed again, and while the reviewers had largely positive comments, they have a few very minor critiques that we hope you can address in a minor revision of the manuscript. Therefore, I invite you to respond to the reviewers' comments and revise your manuscript. The reviewers' comments are included at the bottom of this message.

In addition to the reviewers' comments, please also address our Scientific Editor's comments added below to comply with our requirements for presentation of scientific data at Brain Communications.

Please format your revised manuscript according to our Instructions to Authors https://academic.oup.com/braincomms/pages/General_Instructions – a summary of key points is attached to help with this.

To revise your manuscript, click the link below or log into https://mc.manuscriptcentral.com/braincom and enter your Author Centre, where you will find your manuscript title listed under "Manuscripts with Decisions." Under "Actions," click on "Create a Revision." Your manuscript number will be appended to denote a new revision.

*** PLEASE NOTE: This is a two-step process. After clicking on the link, you will be directed to a webpage to confirm. ***

https://mc.manuscriptcentral.com/braincom?URL_MASK=6f2f8179d9294b61a025f3c2547cd770

Please upload a revised manuscript with changes tracked using track changes or in coloured text.

When submitting your revised manuscript, you will be asked to respond to the reviewer(s) comments. Please be as specific as possible in your response to all of the comments.

The editorial team at Brain Communications also aims to increase transparency in scientific publishing and to demonstrate the value that peer review brings to the scientific process. Please therefore consider whether you are willing to have reviewer comments on your paper and your response published as supplementary data with the paper if it is accepted. This is entirely optional and will only be done if both the authors and reviewers agree. When submitting your revision you will have the opportunity to update the answer you entered previously.

IMPORTANT: Your original files are available to you when you upload your revised manuscript. Please delete any redundant files before completing the submission.

Because we are trying to facilitate timely publication of manuscripts submitted to Brain Communications, we would appreciate your revised manuscript as soon as possible. If it is not possible for you to resubmit within 3 months, the option to submit a revision will expire, and we would then consider your revised paper as a new submission. However, if you need a little more time, please get in touch and we can extend this deadline for you.

Once again, thank you for submitting your manuscript to Brain Communications and I look forward to receiving your revision.

Best wishes

Dr. Alberto Lleo
Associate Editor, Brain Communications

Professor Tara Spires-Jones
Founding Editor, Brain Communications

Reviewer(s)' Comments to Author:

Reviewer: 1

Comments to the Author
The authors have addressed all my comments in convincing detail.
Minor suggestion: the coefficient and SE columns in table 2 could be presented in scientific notation.

Reviewer: 2

Comments to the Author
I thank the authors for their answers. I am overall happy with the changes made and only have three remaining questions/suggestions.

1. Title. The authors state that their first aim was to "determine whether low gestational age at birth was associated with variation in the salivary methylome". This is now not reflected in the title. I would suggest something like this: "DNA methylation in relation to gestational age and brain dysmaturation in preterm infants".
2. Potential causes of prematurity in the current study. I am aware that the cause of preterm birth is often unknown and usually multi-factorial and that it is not possible to state the cause of preterm birth with certainty for participants in your cohort study. Yet, it would be informative to add at least some information about potential causes to Table 1. For example, the prevalence of maternal diabetes mellitus, hypertension, pre-eclampsia, infection might be available. Could the authors add such maternal characteristics to table 1 if available?
3. Prevalence of maternal smoking. The authors mention that the percentage of smoking mothers in the preterm group (19%) is higher than the estimated 14.6% of the general population in Scotland. Reading the authors' answer, I realized that the slightly higher percentage of smoking mothers in the preterm group is in fact not the main noticeable point – but rather the low smoking prevalence of only 2% in mothers of term born infants. These results suggest a potential selection towards a healthy population from higher socio-economic background in the term group. Could the authors comment on this in the discussion? Also, it would be informative to add maternal educational level to Table 1.

Comments from the Scientific Editor:

- Please, include the quantitative parameters related to the analysis included in the figures in the corresponding legends.
