# Supplementary material for: DNA methylation in relation to gestational age and brain dysmaturation in preterm infants
Source: Brain Commun. 2022 Mar 8;4(2):fcac056. doi: 10.1093/braincomms/fcac056 (PMC8984700; doi:10.1093/braincomms/fcac056)
Supplement: fcac056_Supplementary_Data [file Revision 1 _Authors Response.pdf › Revision 1 _Authors Response.pdf]

Reviewer: 1

Comments to the Author
The authors have addressed all my comments in convincing detail.
Minor suggestion: the coefficient and SE columns in table 2 could be presented in scientific notation.

Thank you. We have now addressed the remaining minor suggestion in Table 2 and Table 3.

Reviewer: 2

Comments to the Author
I thank the authors for their answers. I am overall happy with the changes made and only have three remaining questions/suggestions.

1. Title. The authors state that their first aim was to “determine whether low gestational age at birth was associated with variation in the salivary methylome”. This is now not reflected in the title. I would suggest something like this: “DNA methylation in relation to gestational age and brain dysmaturation in preterm infants”.

Response 2. Thank you for the positive comment about our first revision. We have changed the title as per this request.

2. Potential causes of prematurity in the current study. I am aware that the cause of preterm birth is often unknown and usually multi-factorial and that it is not possible to state the cause of preterm birth with certainty for participants in your cohort study. Yet, it would be informative to add at least some information about potential causes to Table 1. For example, the prevalence of maternal diabetes mellitus, hypertension, pre-eclampsia, infection might be available. Could the authors add such maternal characteristics to table 1 if available?

Response 3. We have added information about maternal diabetes, hypertension, and pre-eclampsia to Table 1. We do not have information about maternal infection for this cohort. We have added information about maternal education to the Table (see response 4).

3. Prevalence of maternal smoking. The authors mention that the percentage of smoking mothers in the preterm group (19%) is higher than the estimated 14.6% of the general population in Scotland. Reading the authors’ answer, I realized that the slightly higher percentage of smoking mothers in the preterm group is in fact not the main noticeable point – but rather the low smoking prevalence of only 2% in mothers of term born infants. These results suggest a potential selection towards a healthy population from higher socio-economic background in the term group. Could the authors comment on this in the discussion? Also, it would be informative to add maternal educational level to Table 1.

Response 4. Thank you. The reviewer is correct that the term group come from a higher socioeconomic background. This is indicated by a higher proportion of mothers with college/university qualifications compared to high school or no qualifications in the term group, compared to the preterm group. We have added this education information to Table 1 as requested. In this sense, the study cohort is representative because preterm birth itself follows a social gradient. We agree this is an interesting point to discuss and have added the following (page 14):

“Finally, preterm birth itself can reflect maternal health or circumstance; in this cohort mothers of preterm infants were more likely to have pregnancy-induced hypertension and the control mothers were more likely to have college or university qualifications. It is possible that these, or other maternal factors associated with preterm birth such as infection, chronic disease, poor nutrition, and alcohol, tobacco or drug use, could influence the neonatal methylome at term equivalent age. A large prospective study with longitudinal sampling from birth would be required to test the hypothesis that maternal factors contribute to changes in the methylome apparent after neonatal intensive care.”

Comments from the Scientific Editor:

- Please, include the quantitative parameters related to the analysis included in the figures in the corresponding legends.

Response 5. We have included parameters for all variables in the legends.
